# Neuroprotective Effects of Bexarotene and Icariin in a Diabetic Rat Model

**DOI:** 10.7759/cureus.68238

**Published:** 2024-08-30

**Authors:** Dilek Agircan, Tugba Melike Parlak, Oznur Tufan, Muhammed Demircioglu, Burak Dik

**Affiliations:** 1 Department of Neurology, Faculty of Medicine, Harran University, Sanlıurfa, TUR; 2 Department of Pharmacology and Toxicology, Faculty of Veterinary Medicine, Selcuk University, Konya, TUR; 3 Department of Histology and Embryology, Institute of Health Sciences, Dicle University, Diyarbakir, TUR

**Keywords:** neuroprotective agents, icariin, bexarotene, neurodegenerative diseases, type 2 diabetes mellitus (type 2 dm)

## Abstract

Objective

Type 2 diabetes mellitus (T2DM), a chronic metabolic disorder affecting over 400 million people globally, is increasingly recognized for its detrimental impact on the central nervous system. T2DM is linked to neurodegenerative diseases like Alzheimer's and vascular dementia. This study investigates the neuroprotective effects of bexarotene and icariin in a T2DM rat model, focusing on brain-derived neurotrophic factor (BDNF), glial fibrillary acidic protein (GFAP), and neurofilament-light chain (NfL) levels.

Methods

Before the study, rats underwent fasting blood glucose tests, lipid profile assessments, and general health evaluations, followed by a high-fat diet for two weeks and a single streptozotocin dose (35 mg/kg). Rats with fasting blood glucose levels ≥250 mg/dl were classified as diabetes mellitus (DM) and continued on the high-fat diet throughout the experiment. Forty-seven male Wistar Albino rats were divided into six groups: a healthy control group, a DM control group, a DM group treated with bexarotene, a DM group treated with icariin, and two DM groups treated with combinations of low and high doses of bexarotene and icariin. After the 45-day treatment, blood samples were collected under thiopental sodium anesthesia, with HbA1c (glycosylated hemoglobin) and hematological parameters analyzed within eight hours, and serum stored at -80°C for further analysis. The animals were then euthanized, and brain tissues were harvested, frozen, and stored at -80°C until further examination. Brain tissues were analyzed for BDNF, GFAP, and NfL levels using ELISA (enzyme-linked immunosorbent assay). For comparing multiple groups, the Kruskal-Wallis test was applied to nonparametric data, and one-way ANOVA was used for parametric data, followed by Bonferroni's post hoc test for pairwise comparisons. Statistical significance was determined with two-tailed tests at p < 0.05.

Results

Significant changes in GFAP levels were observed across groups (p < 0.001). The DM control group showed the highest GFAP levels, while treatment groups exhibited reductions. The DM control group also showed the highest BDNF levels, while treatment groups exhibited reductions. The DM control group showed the lowest NfL levels, while treatment groups exhibited increments.

Conclusion

This study highlights the neuroprotective potential of bexarotene and icariin in a diabetic rat model, evidenced by significant changes in GFAP levels. The lack of significant changes in BDNF and NfL suggests that longer study durations may be necessary to observe these effects. Future research should include extended study periods, larger sample sizes, varied dosages, and comprehensive behavioral assessments to better understand the therapeutic potential of these agents.

## Introduction

Type 2 diabetes mellitus (T2DM), a chronic metabolic disorder characterized by persistent hyperglycemia, has been increasingly acknowledged for its destructive effects on the central nervous system (CNS) [1]. The International Diabetes Federation estimates that over 400 million individuals worldwide are affected by T2DM, making it one of the most widespread global epidemics [2]. Recent studies have marked up a strong association between diabetes mellitus (DM) and a high risk of neurodegenerative diseases, such as Alzheimer’s disease (AD) and vascular dementia [3]. The mechanisms underlying this association are varied, involving a complex interaction of hyperglycemia, insulin resistance, inflammation, and microvascular complications [4]. Hyperglycemia-induced oxidative stress and the formation of advanced glycation end products contribute to neuronal damage and synaptic dysfunction [5]. Oxidative stress has been proposed as a contributing factor in the development of AD for a considerable time [6]. Furthermore, insulin resistance impairs insulin signaling pathways in the brain, which are critical for cognitive functions and neuronal survival [7]. In addition, dysregulation of the mTOR (mammalian target of rapamycin) signaling pathway is implicated in both AD and DM [8].

Diabetes mellitus reduces brain-derived neurotrophic factor (BDNF) levels by enhancing oxidative stress or through other independent mechanisms, indicating that restoring the redox balance in DM may elevate BDNF levels and prevent associated complications and neurodegenerative diseases [9]. Numerous studies have emphasized that glial fibrillary acidic protein (GFAP) protein levels decrease in the cerebellum, cerebral cortex, and hippocampus of diabetic animals [10,11]. Neurofilament-light chain (NfL) levels are strongly associated with cognitive performance in diabetic mouse models [12].

Icariin, a flavonoid obtained from the traditional Chinese herb *Epimedium brevicornum* Maxim, exhibits numerous pharmacological and biological effects. Icariin exhibits multiple neuroprotective effects in various models of neurodegenerative diseases, particularly AD. It enhances the NO/cGMP pathway in amyloid-beta (Aβ) APP/PS1 transgenic mice, promoting vascular function. Icariin also upregulates the BDNF-TrkB-ERK/Akt pathway, improving hippocampal neural stem cell function in Aβ-induced models. Additionally, it reduces Tau protein hyperphosphorylation via the MAPK pathway in rat cortical neurons and supports neuronal survival by activating the PI3K/Akt/GSK-3β pathway in neuronal cells. Furthermore, icariin inhibits mitochondrial dysfunction and neuroinflammation by modulating MAPK, NF-κB, and ER stress pathways in various models, including IBO-injected rats and APP/PS1 mice. It also regulates synaptic structure and neurotransmitter function through the BDNF/TrkB/Akt pathway in Aβ-induced rats and improves mitochondrial function in AD mice by balancing key mitochondrial proteins [13,14]. It offers anti-tumoral impacts, antioxidant capabilities, immune system modulation, and neuroprotective properties [15]. Clinical trials have been conducted on these compounds to treat AD, revealing beneficial outcomes in patients with the condition [16]. Additionally, it inhibits the accumulation of pathogenic AD proteins, including Aβ deposits and hyperphosphorylated tau [17]. It reduced the severity of DM by protecting pancreatic islet count and function in a diabetic rat model [18]. Treatment with icariin preserves neuronal and synaptic integrity by counteracting impaired brain insulin signaling and glucose hypometabolism.

Bexarotene primarily exerts its effects through activating retinoid X receptors (RXRs), which are nuclear receptors involved in regulating gene expression related to cell differentiation, proliferation, and apoptosis. By acting as an RXR agonist, bexarotene influences various cellular processes, including the clearance of amyloid-beta in neurodegenerative diseases such as AD. This mechanism is crucial for reducing the amyloid plaque burden, a hallmark of AD's pathology. Additionally, bexarotene modulates lipid metabolism by activating liver X receptors, which play a significant role in regulating cholesterol and phospholipid homeostasis. This modulation further contributes to the reduction of amyloid-beta accumulation in the brain. In the context of oncology, bexarotene induces apoptosis in certain cancer cells, such as those in cutaneous T-cell lymphoma, by promoting the expression of pro-apoptotic genes and inhibiting cellular proliferation. Furthermore, bexarotene exhibits anti-inflammatory properties, which may provide additional therapeutic benefits in both neurodegenerative diseases and cancer [19,20].

The present study aims to examine the neuroprotective properties of bexarotene and icariin in T2DM. We hypothesized that bexarotene and icariin would exhibit neuroprotective effects in the streptozotocin (STZ) induced T2DM model by modulating BDNF, GFAP, and NfL levels in brain tissue.

## Materials and methods

Animal subjects

This research utilized 47 male Wistar Albino rats, aged 8-12 weeks and weighing approximately 250 grams. Before inclusion in the study, the rats underwent fasting blood glucose testing, basal lipid profile assessment, and general health evaluations. The rats were housed with unrestricted access to food and water. The diet composition was as follows: dry matter: 89%, crude protein: 21%, cellulose: maximum 5%, ash: maximum 10%, Ca: 1-2%, P: 0.5-1%, NaCl: 0.5%, and metabolizable energy: 2850 kcal/kg.

Establishment of the experimental type 2 diabetes mellitus model

The T2DM model was established according to protocols described by Srinivasan et al. and Soetikno et al. [21,22]. In this procedure, the rats were fed a high-fat diet for two weeks and a single low dose of STZ (35 mg/kg, subcutaneously) was administered at the end of the second week. Seventy-two hours after STZ administration, animals with a fasting blood glucose level of ≥250 mg/dl were classified as DM. These DM rats continued on the high-fat diet for the duration of the experiment.

Formation of experimental groups

**Figure 1:**
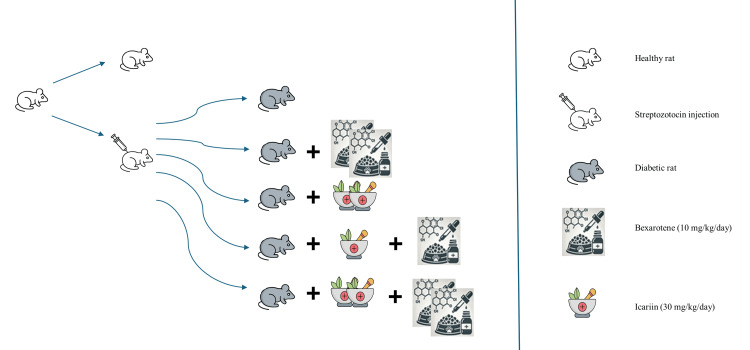
Experimental design and group formation Image credit: Dilek Agircan

The environmental conditions for housing the rats were strictly controlled, with a 12/12-hour light/dark cycle (7:30/19:30), temperature maintained at 22±2°C, and humidity at 55±5%. Out of the 47 rats, 40 were induced with the experimental DM model and divided into five groups, while the remaining seven rats served as the healthy control group (Figure 1).

The healthy control group, consisting of seven rats, was provided with standard rat chow and water ad libitum and received physiological saline (4 ml/kg/day, orally) concurrent with treatments given to other groups. The DM control group included eight rats with confirmed DM, verified by blood glucose measurement, which received physiological saline (4 ml/kg/day, orally) for 45 days. In the DM + bexarotene group, eight DM rats were administered bexarotene (20 mg/kg/day, dissolved in corn oil, orally) for 45 days. Another group, the DM + icariin group, comprised eight DM rats that received icariin (60 mg/kg/day, dissolved in olive oil, orally) for 45 days. The DM + low dose combination group included eight DM rats that were administered both bexarotene (10 mg/kg/day, dissolved in corn oil, orally) and icariin (30 mg/kg/day, dissolved in olive oil, orally) for 45 days. Lastly, the DM + high dose combination group consisted of eight DM rats given bexarotene (20 mg/kg/day, dissolved in corn oil, orally) and icariin (60 mg/kg/day, dissolved in olive oil, orally) for 45 days (Figure 1). Based on prior preclinical studies, the doses of bexarotene [23] and icariin [17] were selected for this research.

After the 45-day treatment period, blood samples were collected from the heart under thiopental sodium anesthesia (40 mg/kg, intraperitoneally) into ethylenediaminetetraacetic acid (EDTA)-coated gel tubes. The blood samples collected in EDTA tubes were analyzed for HbA1c (glycosylated hemoglobin) using an HbA1c analyzer (Trinity Biotech, Premier Hb9210, Ireland) and for hematological parameters (WBC, RBC, hematocrit, platelets) using a hematology analyzer (Mindray Bio-Medical Electronics, Shenzhen, China) within eight hours. Blood collected in gel tubes was centrifuged at 4000 rpm, and the resulting serum was aliquoted into Eppendorf tubes and stored at -80°C for further analysis.

After the 45-day treatment period, the animals were euthanized by thiopental Na anesthesia (40 mg/kg, IP), and the brain tissues were harvested and frozen into the cryo tubes by nitrogen. The tissues were stored at -80°C until analysis.

ELISA analyses

The brain tissues were homogenized by homogenizer (Heidolph, Silent Crusher M, Germany) and determined BDNF (Rat BDNF ELISA {enzyme-linked immunosorbent assay} kit, Cat no: E0476Ra, Bioassay Technology Laboratory, Shangai, China), NfL (Rat NfL ELISA kit, Cat no: E2549Ra, Bioassay Technology Laboratory, Shangai, China), GFAP (Rat GFAB, ELISA kit, Cat no: E0538Ra, Bioassay Technology Laboratory, Shangai, China) levels were analyzed using rat-specific commercial ELISA kits. The analyses were conducted according to the procedures specified in the kits.

Ethical considerations

All experimental procedures involving animals were conducted following the guidelines for the care and use of laboratory animals and were approved by Harran University (Protocol Number: 2024/004/07). The welfare of the animals was a primary concern, and all efforts were made to minimize suffering and distress.

Statistical analyses

Statistical analyses were conducted using IBM SPSS Statistics for Windows, Version 24, (IBM Corp., Armonk, NY) The normality of data distribution was assessed using the Shapiro-Wilk test. Numerical variables that followed a normal distribution were presented as means ± standard deviations, whereas those not conforming to normal distribution were described using median and interquartile range values. While comparing multiple groups Kruskal-Wallis test was used for nonparametric data and one-way analysis of variance (ANOVA) for parametric data, followed by Bonferroni's post hoc test for pairwise comparisons. The Spearman rank correlation was utilized for the correlation analysis. All statistical tests were two-tailed, with a significance level set at p < 0.05.

## Results

There were seven rats in the healthy control group and eight rats in each of the other groups. BDNF levels were significantly different among the groups (p < 0.001). The healthy control group showed a mean BDNF level of 1.63 (0.5). The DM control group had a slightly higher mean BDNF level of 1.77 (1.678), but this increase was not statistically significant. In contrast, the DM + bexarotene group exhibited a significantly lower BDNF level of 0.775 (0.878) compared to the DM control group, and the DM + icariin group showed a similarly reduced level of 0.78 (0.52), which was also statistically significant. The DM + low dose combination group had a BDNF level of 0.83 (0.648), while the DM + high dose combination group showed an intermediate BDNF level of 1.095 (0.715). While the DM + high dose combination group's BDNF level was not significantly different from the healthy control group, it was higher than the other treatment groups, suggesting a potential dose-dependent effect that may warrant further investigation. GFAP levels also showed significant variation among the groups (p < 0.001). The healthy control group had a mean GFAP level of 340.99 (141.08). The DM control group exhibited a higher GFAP level of 396.12 (46.24), but this increase was not statistically significant. The DM + bexarotene group and the DM + icariin group had statistically significantly lower GFAP levels compared to the DM control group with 172.055 (90.4) and 169.68 (81.798), respectively. Similarly, the DM + low dose combination group showed a GFAP level of 166.305 (43.573), whereas the DM + high dose combination group had a level of 292.98 (124.485). The GFAP levels in the DM + high dose combination group were not significantly different from the DM control group. NfL levels were also significantly different across the groups (p < 0.001). The healthy control group had a mean NfL level of 157.39 (± 78.992). The DM control group had a similar NfL level of 154.425 (± 40.03); this increase was not statistically significant. The DM + bexarotene group had a significantly higher NfL level of 264.523 (± 78.247), while the DM + icariin group showed an even greater NfL level of 330.843 (± 105.125), both of which were statistically significant. The DM + low dose combination group had an NfL level of 328.214 (± 58.112), whereas the DM + high dose combination group showed a level of 239.405 (± 98.357); however, these differences were not statistically significant. Groups that do not exhibit statistically significant differences are denoted by the same letter in Table 1.

**Table 1:** Levels of BDNF, GFAP, and NfL across different experimental groups in DM rat model ^a,b,c^No significant differences were observed between groups that were denoted by the same letter *ANOVA, #Kruskal-Wallis test SD = standard deviation, DM = diabetes mellitus, BDNF = brain-derived neurotrophic factor, GFAP = glial fibrillary acidic protein, NfL = neurofilament-light chain

	Healthy control	DM control	DM + bexarotene	DM + icariin	DM + low dose combination	DM + high dose combination	p
	n=7	n=8	n=8	n=8	n=8	n=8	
BDNF, mean (SD)#	1.63 (0.5)^c^	1.77 (1.678)^bc^	0.775 (0.878)^ac^	0.78 (0.52)^a^	0.83 (0.648)^a^	1.095 (0.715)^abc^	<0.001
GFAP, mean (SD)#	340.99 (141.08)^ab^	396.12 (46.24)^b^	172.055 (90.4)^a^	169.68 (81.798)^a^	166.305 (43.573)^a^	292.98 (124.485)^ab^	<0.001
NfL, mean (SD)*	157.39 ± 78.992^c^	154.425 ± 40.03^bc^	264.523 ± 78.247^abc^	330.843 ± 105.125^a^	328.214 ± 58.112^a^	239.405 ± 98.357^abc^	<0.001

**Figure 2:**
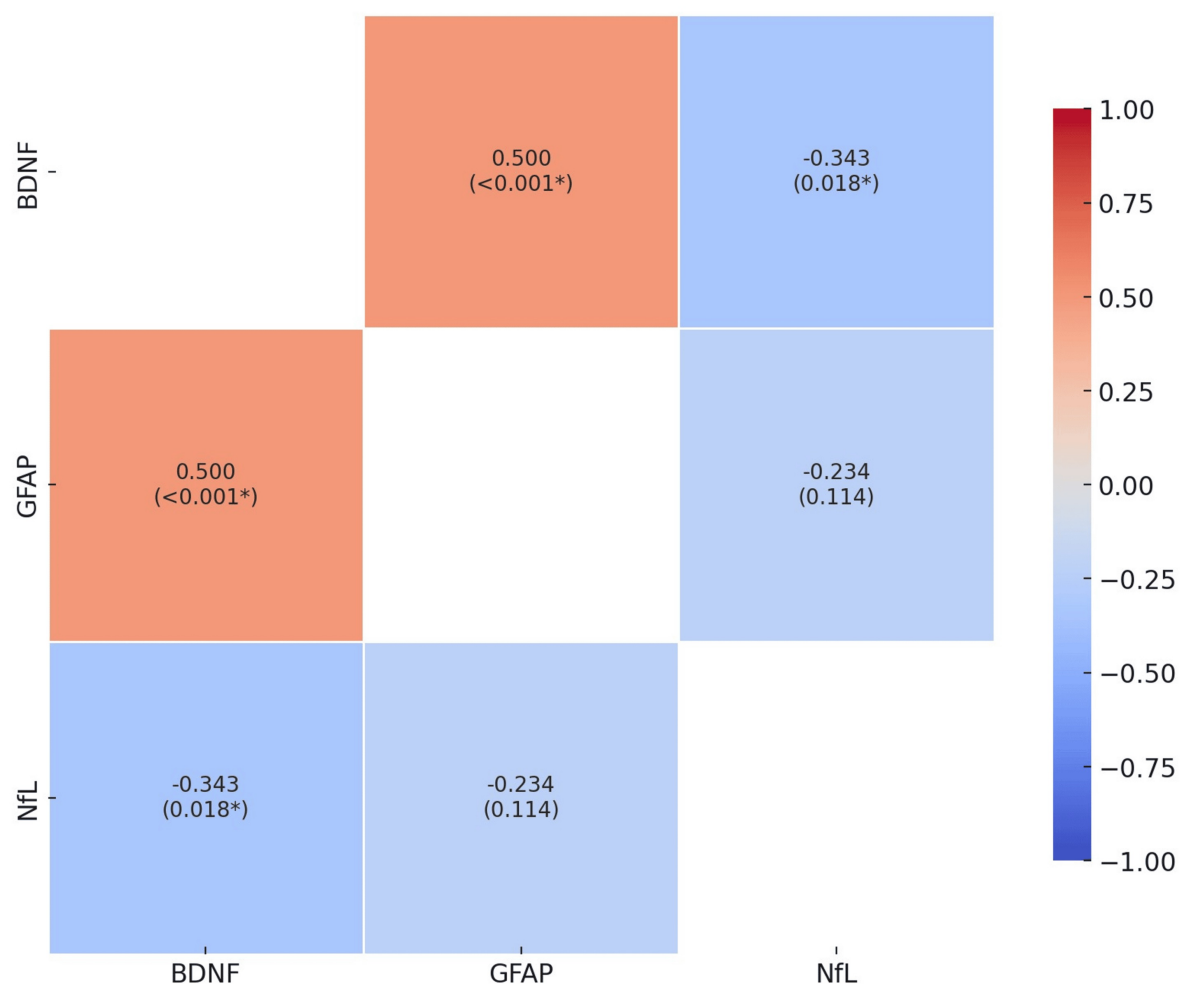
Correlation matrix of BDNF, GFAP, and NfL levels BDNF = brain-derived neurotrophic factor, GFAP = glial fibrillary acidic protein, NfL = neurofilament-light chain *statistical significance at p < 0.05

Figure 2 illustrates the correlation between the levels of BDNF, GFAP, and NfL. There is a statistically significant correlation between BDNF and GFAP, with a positive correlation of 0.500 (p < 0.001), and between BDNF and NfL, with a negative correlation of -0.343 (p = 0.018), indicating that higher BDNF levels are associated with increased GFAP and decreased NfL levels.

## Discussion

The impact of DM on the central nervous system has attracted significant interest, manifesting as cognitive impairment and neurophysiological alterations in the brain. Numerous studies have demonstrated that diabetic patients face a higher relative risk of developing dementia [24]. Recent research has highlighted the popularity of bexarotene and icariin in dementia studies, given their promising therapeutic potential in this area [15,20].

Streptozotocin, also referred to as streptozocin, was first identified in 1959 as a natural antibiotic synthesized by *Streptomyces achromogenes*. In 1963, its selective toxicity to pancreatic β-cells was reported, demonstrating a diabetogenic effect. The STZ molecule consists of two critical components: a glucopyranosyl group, which facilitates its uptake into pancreatic β-cells through the glucose transporter GLUT2, and a nitrosourea group, which triggers the destruction of these β-cells. These elements cause a reduction in insulin production and secretion, ultimately leading to hyperglycemia, a hallmark of diabetes [25]. The susceptibility to AD is closely associated with impaired insulin sensitivity. Intracerebroventricular (ICV) and intraperitoneal (IP) injections of STZ-induced neuroinflammation and cognitive impairments in rodent models. Systemic STZ administration primarily targets pancreatic beta cells via oxidative stress and DNA damage pathways, whereas ICV administration predominantly impacts the brain, exerting secondary effects on beta cell function. Notably, bilateral ICV administration of STZ in mice results in persistent neuroinflammation, along with elevated levels of Aβ and hyperphosphorylated tau (p-tau) within the hippocampus. Therefore central ICV administration of STZ is considered a more accurate model of sporadic AD compared to peripheral STZ administration [26,27].

In diabetic patients and animal models, glucose metabolism in the brain is profoundly affected. The transport of glucose, glycolysis, the pentose phosphate pathway, and the tricarboxylic acid cycle are all disrupted. These abnormalities lead to decreased adenosine triphosphate synthesis and increased oxidative stress and inflammation. Consequently, this cascade results in reduced synthesis of neurotransmitters and their modulators, disrupted synaptic plasticity, and ultimately neuronal damage and cognitive impairment [28]. Hippocampal neurons in both diabetic patients and animal models exhibit significant changes, such as synaptic structure damage, axonal degeneration, and neuronal loss [29]. Bexarotene stimulates pathways involved in neurodevelopment and plasticity in adult mice brains [30] and has demonstrated neuroprotective roles in various CNS conditions, such as subarachnoid hemorrhage (SAH), traumatic brain injury (TBI), and thromboembolic stroke [31-33]. In a murine model of amyotrophic lateral sclerosis, administration of bexarotene was found to decrease neuronal mortality while enhancing overall survival rates [34]. Early and pronounced effects of once-daily bexarotene treatment on neural network excitability were observed in two epilepsy models [35]. McFarland et al. observed significant neuroprotective effects of bexarotene in a rodent Parkinson's disease model, primarily through the activation of the Nurr1 receptor and its downstream genes, such as CREB and BDNF [36]. These results are in agreement with recent studies indicating that the activation of PPARα by bexarotene promotes BDNF expression and enhances memory in an AD mouse model [37]. It has been shown that icariin may enhance the proliferation and activation of astrocytes, crucial for neural environment support and maintenance, as evidenced by the rise in GFAP-positive cells [38]. In a mouse model of TBI, icariin alleviated cognitive impairment by promoting hippocampal acetylation [39]. In a neonatal rat model of epilepsy induced by hypoxia, icariin enhanced cognitive function and also provided neuroprotection [40]. Icariin not only improved post-stroke dementia but also exhibited a neuroprotective effect on dopaminergic neurons in a Parkinson's disease mouse model [15,41]. In C57BL/6J mice, icariin treatment alleviated cuprizone-induced demyelination by enhancing BDNF expression, which promoted oligodendrocyte differentiation and reduced the presence of microglia and astrocytes in the affected brain regions [42]. Icariin has been shown to significantly enhance learning and memory in various AD models through mechanisms such as beta-secretase inhibition, antioxidative stress, anti-inflammatory effects, and the upregulation of BDNF [43]. In light of these findings, our results showed a significant change in GFAP levels following treatment, suggesting potential neuroprotective effects in the present study in which we focused on the impact of bexarotene and icariin treatments on neurodegeneration markers in diabetic mice. However, no significant changes were observed in BDNF and NfL levels. We believe that the lack of increase in neurodegeneration-related molecules in our study may be due to the peripheral STZ administration being insufficient to establish an Alzheimer's model and the shorter duration of the DM model.

Astrocytes are pivotal in maintaining glycogen reserves within the brain and play a crucial role in maintaining neuronal survival and function by regulating the ionic environment in the brain. In the initial stages of DM, astroglial activation can be detected, whereas extended periods of DM may lead to astroglial cell death. Astroglial responses can differ based on the severity of DM and the specific subtypes of astroglia involved [11]. Astrocytes increase GFAP production, a process known as reactive gliosis, which is essential for the morphogenesis of the central nervous system [44]. In our study, we suggest that the increase in GFAP observed in the diabetic control group compared to the healthy control group might be attributed to the shorter duration of DM.

In recent studies, BDNF has been identified as a key regulator of synaptogenesis and synaptic plasticity, which are essential for learning and memory processes in the adult central nervous system. Furthermore, BDNF aids in the differentiation, growth, target innervation, and survival of neurons throughout the development of both the central and peripheral nervous systems [45]. NfL is highly expressed in axons, as well as dendrites and the neuronal soma, and provides structural stability to neurons. Its levels rise in various neurological disorders, including inflammatory, neurodegenerative, traumatic, and cerebrovascular diseases, increasing in proportion to the degree of axonal damage [46]. Astrocytes demonstrate the initial cellular reactions by markedly increasing GFAP expression after an injury to the central nervous system [47]. Compared to neurons, glial cells exhibit a markedly heightened vulnerability to neurotoxic insults and demonstrate a significantly amplified sensitivity to neuroprotective therapies [48]. In a study conducted on a mouse model, brain tissue damage was evaluated at eight and 12 weeks after STZ injection, revealing that the brain tissue damage was less severe at eight weeks compared to 12 weeks [49]. Given this sensitivity, the lack of significant changes in BDNF and NfL despite the changes observed in GFAP following treatment in our study may be attributed to the more sensitive astrocyte-derived GFAP compared to the less sensitive neuron-derived BDNF and NfL, as well as the relatively short six-week duration of our study. We hypothesize that BDNF and NfL will show more significant changes in longer-term studies due to both disease progression and extended therapeutic interventions.

Limitations

This study has several limitations that should be considered when interpreting the results. The relatively short duration of the study, spanning only six weeks, may have been insufficient to observe significant changes in diabetic neurodegeneration. Furthermore, the study focused solely on molecular changes, excluding behavioral and functional tests, which are crucial for evaluating changes in neurological and cognitive functions in treated animals. Their exclusion was necessitated by the pre-established scope and parameters of the tissue sample collection.

## Conclusions

Our study highlights the neuroprotective potential of bexarotene and icariin in a diabetic mouse model, with significant changes in glial fibrillary acidic protein (GFAP) levels suggesting astrocyte activation and potential neuroprotective effects. These findings emphasize the sensitivity of GFAP as a marker of early neurotoxic responses, underscoring the critical role of astrocytes in diabetic neurodegeneration. Although brain-derived neurotrophic factor (BDNF) and neurofilament-light chain (NfL) levels did not show significant changes, this may reflect the complexity of neurodegenerative processes and the specific dynamics of neuron-derived markers, which may require longer durations to exhibit measurable alterations. Overall, our results support the therapeutic potential of bexarotene and icariin, particularly in targeting astrocyte-related pathways in diabetic neurodegeneration. While our results are promising, future research should include longer-term studies, larger sample sizes, varied dosages, and comprehensive behavioral assessments.
